# Equitable realization of the right to health in Haiti: how household data inform health seeking behavior and financial risk protection

**DOI:** 10.1186/s12939-019-0973-7

**Published:** 2019-05-27

**Authors:** Marion Cros, Eleonora Cavagnero, Jean Patrick Alfred, Mirja Sjoblom, Nicolas Collin, Tania Mathurin

**Affiliations:** 1 World Bank, Health Nutrition and Population (HNP), 1850 I St NW, Washington, DC, 20006 USA; 2 Director of Research and Planning Unit, Ministry of Public Health and Population of Haiti, Port-au-Prince, Haiti

**Keywords:** Health seeking behavior, Catastrophic health expenditures (CHE), Inequalities, Disaster relief

## Abstract

**Background:**

Though the right to health is included in Haiti’s constitution, little progress has been made to expand universal health coverage nationwide, a strategy to ensure access to health services for all, while preventing financial hardship among the poor. Realizing universal health coverage will require a better understanding of inequities in health care utilization and out-of-pocket payments for health. This study measures inequality in health services utilization and the determinants of health seeking behavior in Haiti. It also examines the determinants of catastrophic health expenditures, defined by the Sustainable Development Goal Framework (Indicator 3.8.2) as expenditures that exceed 10% of overall household expenditures.

**Methodology:**

Three types of analysis were conducted using the 2012 and 2013 Household Surveys (Enquête sur les Conditions de Vie des Ménages Après Séisme (ECVMAS I (2012) and ECVMAS II (2013)) to measure: 1) outpatient services as a measure of inequalities using the 2013 Concentration Index; 2) drivers of health seeking behavior using a logistic regression model for 2013; and 3) determinants of catastrophic health expenditures using Seemingly Unrelated Regressions for both 2012 and 2013.

**Results:**

The rate of catastrophic health expenditures increased nationwide from 9.43% in 2012 to 11.54% in 2013. This increase was most notable among the poorest wealth quintile (from 11.62% in 2012 to 18.20% in 2013), yet declined among the richest wealth quintile (from 9.49% to 4.46% during the same period). The increase in the rate of catastrophic health expenditures among the poorest coincides with a sharp decrease in external donor funding for the health sector. Regression analysis indicated that the rich wealth quintiles were less likely than poor wealth quintiles to incur catastrophic health expenditures. Interestingly, households were less likely to incur catastrophic health expenditures when they accessed care from Community Health Workers than when they received care from other types of providers, including public and private health care facilities. This study also shows that Community Health Worker-provided services have a negative concentration index (− 0.22) and are therefore most utilized by poor quintiles. In contrast, both public and private outpatient services had positive concentration indexes (0.05 and 0.12 respectively) and are most utilized by the rich wealth quintiles. Seeking care from traditional healers was found to be pro-poor in Haiti (concentration index of − 0.18) yet was also associated with higher catastrophic health expenditures albeit the coefficient was not significant.

**Conclusion:**

The expansion of universal health coverage in Haiti is evolving in a ‘pro-rich’ manner. Realizing Haiti’s right to health will require a course-correction supported by national policies that protect the poor wealth quintiles from catastrophic health expenditures. Such policies may include Community Health Worker service delivery expansion in underserved areas. Evidence-based interventions may also be required to lower outpatient user fees, subsidize drug costs and promote efficiencies in pro-poor disaster relief programming.

## Background

The connection between the right to health and Universal Health Coverage (UHC) is unequivocal. The World Health Organization (WHO) defines UHC as access to needed health services for all people while ensuring people do not suffer financial hardship when paying for health services [1]. UHC has been termed as a “practical expression of the right to health” [2]. The human rights-based approach sets clear principles for evaluating health policy and service delivery, targeting discriminatory practices and unjust power relations that perpetuate inequitable health outcomes [3]. By prioritizing the health needs of the poorest, the right to health promotes greater health equity. In turn, this supports developing nations in bridging the disparities between the ability of rich and poor populations to access quality health services, a central goal of Haiti’s UHC agenda. The recent Sustainable Development Goal (SDG) Indicator 3.8.1 (related to population coverage) and SDG Indicator 3.8.2 (the financial dimension of UHC) are tracked by wealth quintile to ensure that the poorer are able to access better health coverage and better financial protection over time [4]. Yet, achieving equitable health systems remains an arduous and allusive goal throughout the developing world.

In the Latin America and Caribbean (LAC) region which is marked by deep social inequalities, 18 countries have explicitly included constitutional rights to health [5] as a means of setting the region on a path to achieving UHC.

Haiti, a country within the LAC region, grapples with a misalignment between the *de jure* right to health and *de facto* inequities that remain in practice. Though the right to health is included and defined in Haiti’s constitution, and there is a renewed commitment to achieving UHC, Haiti has made little progress towards improving health coverage and health outcomes among its poorest wealth quintiles. Despite improvement in the maternal mortality ratio (MMR) (from 625 deaths per 100,000 live births in 1990 to 359 deaths per 100,000 live births in 2013 [6]), and a decline in under-five mortality rate (U5MR) (from 144 deaths per 1000 births to 59 deaths per 1000 births between 1994 and 1995 and 2016 [7]), Haiti continues to suffer some of the poorest health services coverage and outcomes when compared to other countries in the LAC region and other low-income countries (LIC) worldwide. The UHC Service Coverage Index (SCI), which measures the average coverage of essential services, was 48% in Haiti in 2015 [8], slightly higher than that of Sub-Saharan Africa (46%), but much lower than in the rest of LAC (75%) [4].

In Haiti, sharp inequalities in health care service delivery and outcomes between the rich and the poor may be slowing down efforts to improve national health outcomes and coverage indicators. For example, in 2017, 79% of pregnant women in the highest wealth quintile delivered at health facilities compared to 13% in the lowest wealth quintile. Similarly, the vaccination rate was 30% in the lowest wealth quintile compared to 66% among richer households [7]. Globally, households’ out of pocket (OOP) payments for health services, medicines or other medical supplies paid at points of service are known to be a key factor in discouraging the poorest quintiles from seeking preventive and curative health care services [1, 9–11]. Urrutia et al. (2012) report that Haiti reflects this same trend. For example, pregnant women did not use traditional birth attendants (TBAs) or access facility-based health care services due to cost [12]. The 2008 and 2012 Demographic and Health Surveys (DHS) also underscore cost as a key factor in deterring women aged 15–49 years from consulting a health care provider when they are sick.

Lack of affordability is partly linked to the Haitian health care financing system which is highly dependent on both external financing and user OOP payments. For example, in 2015, international donors funded 49% of health expenditures [13], while individual households bore the burden of paying 41% of all health expenditures, a figure that far exceeds the 25% threshold established to protect against financial hardship [9]. Additionally, only 4% of health expenditures are funded through social security funds or other agencies [13], representing a minimal social safety net by any standard. These factors create a complex and highly challenging environment in which Haiti is working to realize the right to health and expand health coverage for the poorest wealth quintiles.

Despite ongoing advocacy for UHC, Haiti’s health care financing model presents great challenges to expanding access to health care services among the country’s most vulnerable populations. Any durable response will require gaining a better understanding of the distribution and root causes of inequality of health service utilization and OOP payments among all wealth quintiles. Beyond the disaggregation of health outcomes and service delivery coverage by income conducted by DHS, the only analysis on inequalities in access to health services and financial protection in Haiti exists within the 2017 World Bank (WB) Health Financing System Assessment [14]. The purpose of this assessment was to examine health care service utilization patterns among wealth quintiles through descriptive statistics.

Building on this WB assessment, this study builds on the referenced WB assessment, and addresses existing research gaps by: 1) estimating inequality in outpatient services among all wealth quintiles, and 2) assessing the determinants of health service utilization and OOP payment for health at the national level. Findings from this study may be utilized to establish evidence-based policies aimed at improving health service coverage and financial protection for Haiti’s poorest wealth quintiles.

## Methodology

### Data source and sampling method

The primary data used to estimate morbidity, health service utilization and CHE rates were obtained from two surveys on living conditions in Haiti conducted in 2012 and 2013 (Enquête sur les Conditions de Vie des Ménages Après Séisme (ECVMAS I and II)) [15]. The 2012 survey, ECVMAS I, had a sample size of 4930 households that was representative at the department and national levels [15]. The 2013 survey, ECVMAS II, was a panel survey with a sample size of 2282 households (which were part of ECVMAS I, including 4930 households). The replacement rate was 8.86%. ECVMAS II included a new module on health, detailed health expenditures and health seeking behavior, which consisted of 21 questions at the individual level)^1^.

### Measurement of inequalities and inequities in outpatient service utilization by provider

This study focuses on outpatient service utilization by provider type, and does not examine inpatient data for the following two reasons: 1) Eight percent of the 2013 ECVMAS II sample measured outpatient services delivered by different types of providers, which allowed for a ‘pro-poor^2^’ assessment of outpatient services by provider type; 2) There were significant gaps in inpatient observation data in ECVMAS II, which constituted less than 3% of the survey sample.

This study utilized the Automated DEC Poverty Tables (ADePT) software developed by the WB [16] to analyze inequalities between wealth quintiles in outpatient health services utilization.^3^ ADePT estimates CI following the procedures described by O’Donnell et al. for micro-data [17]. Inequalities are estimated as the transformation of a variable of interest (e.g., outpatient providers) on fractional rank of wealth within a given population. Outpatient services range from − 1 to 1, representing an accurate distribution from pro-poor to pro-rich health care services.

A detailed decomposition of the CI for outpatient health care service utilization by provider type is presented in Table 5. In this analysis, we differentiate between inequities and inequalities as follows: Inequities refers to the disparity in rates due to differences in social, economic or healthcare resources (i.e., Is the distribution of resources fair?). These are unjustifiable determinants (e.g., wealth, education, health insurance status). In contrast, inequalities refers to how rates vary based on justifiable standardizing determinants such as age and gender (i.e., Can the distribution of outpatient services be influenced by demographic characteristics? [16, 18]).

### Measurement of catastrophic health expenditures (CHE) and health seeking behavior

Defined by the SDG Framework (Indicator 3.8.2) CHE refers to expenditures that exceed 10% of overall household expenditures using a methodology applied to monitor UHC financial protection [4]. This indicator measures the rate of financial hardship incurred by OOP payments. This study defines CHE based on household consumption data (as the 2012 ECVMAS I did not collect income data). In addition, expenditure data is preferable to income data since it is more reliable and considered a better proxy of permanent income [19].

The numerator (total health expenditures) was estimated using survey questions on health spending in the consumption module in both ECVMAS I and II (rather than data collected in the health module in the case of ECVMAS II).This was chosen because respondents tend to report higher expenditures when questions about health expenditures are asked in a separate health module [20]. Health expenditures (e.g., consultations, medicines, hospitalizations, lab work, glasses and prosthesis and other medical supplies) were captured if they were incurred during the ‘last episode of illness’. Households were asked to estimate their expenditures over the previous three and twelve-month period. Evidence showed that longer recall periods yielded lower reported average spending on an annualized basis [21, 22]. Taking into account this limitation, we utilized data collected over the previous 3-month period to capture a more accurate measure of health expenditures.

The denominator was determined by the consumption aggregate created to measure poverty in Haiti (comprised of consumption and non-food expenditures, including health expenditures). We estimated the consumption aggregate to include all types of health expenditures, as the initial consumption aggregate only included recurrent health expenditures (i.e., consultations and medicines). Estimated CHE rates of both truncated and non-truncated data identified minimal differences (i.e., less than 0.5 percentage points) during both survey years.

A health seeking behavior dummy variable^4^ was generated using a question that asked individuals whether they consulted a provider when they were sick over the last 3 months. Affirmative answers were coded as ‘1’ and negative answers were coded as ‘0’. This variable reflected health service utilization.

### Variables selection

Two regression models were utilized for this study. The first regression model examined the determinants of health service utilization in 2013, using the dummy variable for health services utilization as a dependent variable. The second regression model identified the determinants of CHE in 2012 and 2013. The dependent variable was coded as 1 for CHE-affected households, and 0 for households not affected by CHE.

Based on a literature review of the determinants of CHE and health seeking behavior, the independent variables included geography and several household characteristics (e.g., expenditure quintile, household size, education and gender, and having at least one member older than age 65 or younger than age four). The CHE model also included data on provider type (i.e., public, private, CHWs and traditional healers) and health insurance.

Considering OOP payments for health services in the consumption aggregate implied that poor households with substantial health expenditures could shift to a “rich” consumption quintile, even though such expenditures would actually decrease their overall welfare and not make them richer, consumption/expenditure quintile were estimated net of OOP for health [23].

We used ECVMAS I and II to estimate the rate of CHE. However, only 2013 data was available to examine the determinants of health seeking behavior. The 2012 ECVMAS I did not include data on morbidity and health seeking behavior, while the 2013 ECVMAS II examined these issues.

National health expenditures in this study were estimated in Haitian Gourdes (HTG) and geographically geo-deflated^5^.

### Statistical analyses

While health seeking behavior were estimated at the individual level, the rate of CHE was estimated at the household level. The two regression models used a descriptive analysis to identify health utilization and CHE by consumption quintile. Logistic regression was used to predict determinants of health seeking behavior in 2013.

**Table:** 

*Model 1: determinants of health service utilization* Health Utilization 2013 = β0 + β1 wealth quintile +β2 education +β3 urban +β4 region +β5 gender + β6 household size +β7 kid< 4 + β8 old> 65+ u1	

The following variables were used in model 1:

- “Health utilization 2013” is a dichotomous variable (individuals who consulted a provider when sick over the last 3 months are coded 1 and 0 otherwise),
- “Wealth quintile” stands for expenditure quintile and is a dichotomous variable,
- “education” is a dichotomous variable (individuals who went to school are coded 1 and 0 otherwise),
- "urban is a dichotomous variable (individuals who live in urban area are coded 1 and 0 otherwise),
- “region” is a dichotomous variable (individuals who live in the North region are coded 0; in the South, 1; in the Transversal region, 2; in the West, 3 and in the Metropolitan area, 4)
- “gender” is a dichotomous variable (women are coded 1 while men are coded 0),
- “household size” is a continuous variable informing about the number of household’s members,
- “kid< 4” is a dichotomous variable (households with children below 4 years of age are coded 1 and 0 otherwise),
- “old> 65” is a dichotomous variable (households with member(s) above 65 years old are coded 1 and 0 otherwise).

Seemingly Unrelated Regressions (SUR) models were used to estimate the determinants of CHE based on 2012 and 2013 data from each survey year and by applying different explanatory variables. Compared to Ordinary Least Square (OLS), SUR allows dependent variables to have different sets of independent variables [26, 27]. The SUR method simultaneously estimates the parameters of all equations so that the parameters of each single equation also take into account information provided by the other equation. The relationship between these two equations with different independent variables is that the error terms in the two equations can correlate. As a result, SUR may produce more accurate estimates by combining information on different equations rather than running the models separately while allowing joint testing [28].

Two CHE equations (using 2012 and 2013 data^6^) were utilized and run through the SUREG command in STATA 14 [29]. Both the 2012 and 2013 equations were predicted by socio-economic and demographic variables using similar variables to model 1. The following health system variables only available for 2013 were included in the 2013 equation: health insurance, utilization of public and private health facilities, and utilization of CHWs, traditional healers and other ancillary services. Affirmative answers were coded as 1, negative answers were coded with 0. Joint tests utilizing 2012 and 2013 data were also conducted to assess how changes in socio-economic and demographic variables (e.g., wealth quintile, age and household size) effect CHE rates over time.

**Table:** 

*Model 2: Determinants of Catastrophic Health Expenditures* CHE 2012 = γ 0 + γ 1 wealth quintile + γ 2 education + γ 3 urban + γ 4 region + γ 5 gender + γ 6 household size + γ 7 kid< 4 + γ 8 old> 65 + u1 CHE 2013 = γ0 + γ 1 quintile + γ 2 education + γ 3 urban + γ 4 region + γ 5 gender + γ 6 household size + γ 7kid < 4+ γ 8 old> 65 + γ9 public facilities + γ 10 private facilities + γ 11 CHW + γ 12 traditional healers + γ 13 other and ancillary services + γ 14 Health insurance + u1	

## Results

### Descriptive statistics

#### Socio-economic characteristics

Table 1 presents the summary statistics of extracted and computed variables from the 2012 ECVMAS I and 2013 ECVMAS II. The average household size is similar across the 2 years at 6.05 and 6.12, respectively. In each of the survey years, 51.54% and 49.60% of households, respectively, had at least one child under age four. One fifth of surveyed households in both years had an elder aged 65 or older. More than half of the surveyed households were headed by men (57.18% in 2012 and 55.51% in 2013), and slightly fewer than half of the households lived in urban areas. Almost two third of household heads were literate, with a slightly higher proportion in 2013 (65.89%) compared to 2012 (61.58%). The highest concentration of households was in the North, Transversal and the Metropolitan areas of the country (Table 1).

**Table 1:** Descriptive statistics of Models 1 and 2, household level, in Haitian Gourdes (HTG)^a^

Variable description	2012				2013			
	Observation	Proportion	Mean	Standard Deviation (SD)	Observation	Proportion	Mean	Standard Deviation (SD)
Household level	4930				2241			
Household Expenditure	4930		191,976	172,722	2241		204,209	153,315
Rate of Catastrophic Health Expenditures (CHE)	4930		9.43%		2241		11.54%	
Health OOP payments, household level	4930		8091	28,632			19,630	178,073
Health OOP payments-Individual level	4930		1507	5520			3089	33,605
Household size	4930		6.05	2.73			6.12	2.77
Household has under 4-years children	4930	51.54%				49.60%		
Household has elderly	4930	20.16%				20.69%		
Household head is male	4930	57.18%				55.51%		
Household lives in urban area	4930	47.97%				48.35%		
Literate household head	4930	61.58%				65.89%		
Region								
North		20.62%				20.29%		
South		14.74%				13.55%		
Transversal		23.29%				24.73%		
West		19.32%				19.18%		
Metropolitan		22.02%				22.25%		
Households sick the last 30 days					2241	18%		
Households who sought care when sick					2241	76%		
Health Insurance					2241	1.7%		
Households who used outpatient services					2241	18%		
Households who used inpatient services					2241	3%		

Source: ECVMAS I and II (2012 and 2013)

^a^In 2012, 1 USD $ = 42 Haitian Gourdes. In 2013, 1 USD $ = 44 Haitian Gourdes

#### Health seeking behavior

**Fig. 1:**
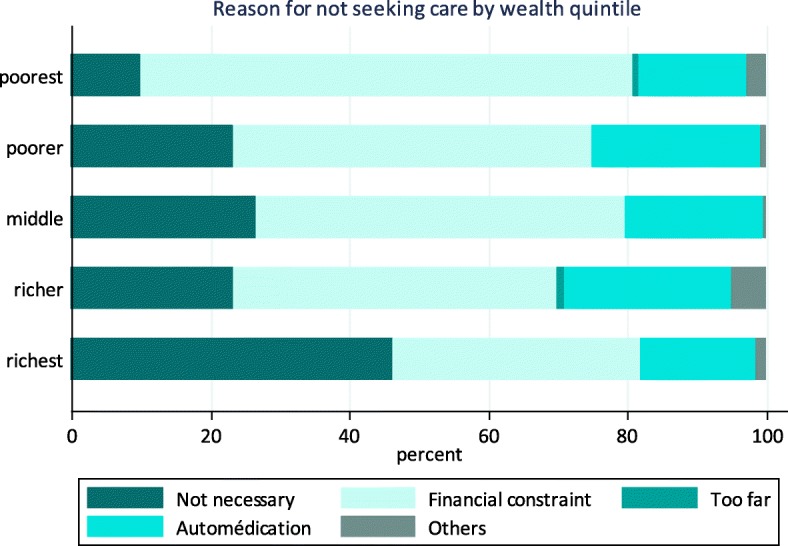
Reasons for not seeking health care by wealth quintile, 2013. Source: ECVMAS II 2013, estimated with wealth quintile net of OOP payments for health at household level

According to the 2013 ECVMAS II, 18% of households sampled reported having an episode of illness in the previous 30 days; and 76% of these households reported having utilized health services during these periods (Table 1). Twenty-four percent of households surveyed did not use health care services. Of these, 56% attributed their decision to the cost of health services (with a higher rate of 70% among poor households compared to 35% among rich households, (Fig. 1)). When family members were sick, 31% of households accessed care through a public hospital, 20% through a public dispensary, 17% through a private provider, 12% through ancillary services (e.g., a pharmacist, drug sellers and laboratories), 7% through CHWs, 6% through a traditional healer and 5% through other service providers.

#### Health expenditures

The mean yearly household expenditure is HTG 191,976 in 2012 and slightly higher in 2013 with HTG 204,209 and the OOP payments healthcare expenditure per household is HTG 8091 in 2012 and HTG 19,630 in 2013 (Table 2). The proportion of households incurring CHE was 9.43% in 2012 and 11.54% in 2013. Since health expenditures have increased at a faster pace than total household expenditures between 2012 and 2013, OOP payments for health as share of total household expenditures increased from 3.42% in 2012 to 4.46% in 2013 (Table 2).

**Table 2:** Household health expenditure characteristics by wealth quintile, household level, 2012 and 2013

	Poorest	Poorer	Middle	Richer	Richest	Mean
2012						
Total household expenditures (THexp)	76,975 (70,425)^a^	126,883 (115,544)^a^	170,640 (158,897)^a^	219,003 (206,179)^a^	366,512 (296,538)^a^	191,976 (151,893)^a^
OOP payments for health (in HTG)	3978 (422)^a^	6587 (1112)^a^	5693 (1644)^a^	7136 (2056)^a^	17,066 (4220)^a^	8091 (1390)^a^
OOP payments for health, % THexp	3.94%	3.64%	2.96%	2.9%	3.68%	3.42%
CHE, 10% THexp	11.62%	10.27%	8.50%	7.27%	9.49%	9.43%
2013						
THexp	97,090 (77,739)^a^	140,174 (134,005)^a^	187,095 (163,182)^a^	243,332 (220,968)^a^	353,562 (294,244)^a^	204,209 (165,993)^a^
OOP payments for health	58,864* (218)^a^	7188 (495)^a^	10,203 (1542)^a^	10,984 (2379)^a^	10,778 (2181)^a^	19,630 (1329)^a^
OOP payments for health, % of THexp	7.99%	4.09%	4.30%	3.38%	2.61%	4.46%
CHE, 10% THexp	18.20%	13.07%	13.52%	9.63%	4.49%	11.54%

^a^ median; top OOP payment spenders were 4 households within the lowest quintiles where they spent between HTG 91,000 – 1,077,000 on health care

Comparison by wealth quintile shows that OOP payments for health as a percentage of total household expenditures increased particularly among the poorest wealth quintiles (from 3.94% in 2012 to 7.99% in 2013), representing a sizable increase of 103%. In contrast, OOP payments for health as a percentage of household expenditures decreased among the richest quintiles from 3.68% in 2012 to 2.61% in 2013 (− 29%) (Table 3). Results for the poorest wealth quintile in 2013 were driven by four households who were the top OOP payment spenders, spending between HTG 91,000 and 1,077,000 on health care. The median, in brackets, shows that 50% of the poorest households only spent HTG 218 per year, compared to HTG 2181 of the richest households. Notably, households affected by CHE increased by 57% in the poorest quintile (from 11.62% in 2012 to 18.20% in 2013), yet fell from 9.49% to 4.49% in the richest quintile.

**Table 3:** Percentage change in household health expenditures between 2012 and 2013

	Poorest	Poorer	Middle	Richer	Richest	Mean
THexp	26%	10%	10%	11%	−4%	6%
OOP payments for health	1380%	9%	79%	54%	−37%	143%
OOP payments for health, % of THexp	103%	12%	45%	17%	−29%	30%
CHE, 10% THexp	57%	27%	59%	32%	−53%	22%

Source: ECVMAS 2012 & 2013

**Fig. 2:**
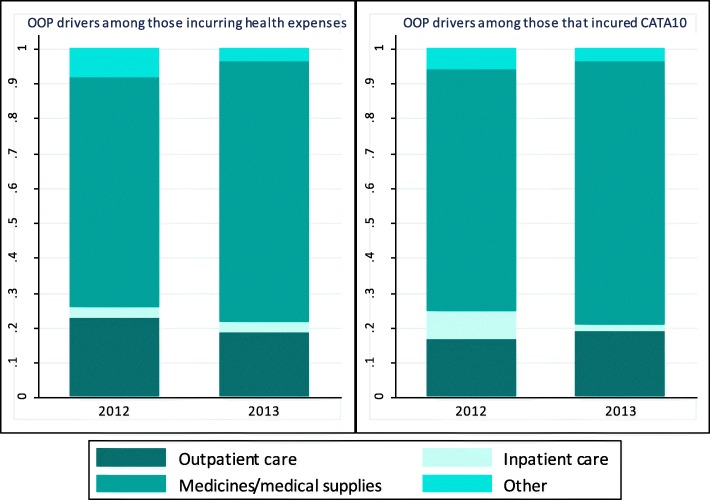
Drivers of health care spending, at the household level, 2012 and 2013. Source: ECVMAS I and II (2012 & 2013); CATA10 is CHE at 10% of household consumption

Health expenditures were examined by type. In both years, medicines and medical supplies were the key drivers of health expenditures among households which incurred CHE. Medicines and medical supplies represented nearly 65% and 70%, respectively, of OOP payments for health care services among households who incurred CHE in 2012. In comparison, these figures rose to 74% and 78% respectively in 2013 (Fig. 2). Utilization of outpatient services was identified as an important driver of CHE (increasing from 16% in 2012 to 19% in 2013), while hospitalization services decreased from 10% to 1% over the same 2 years.

### Econometrics analysis

#### Concentration index (CI) and curve

**Fig. 3:**
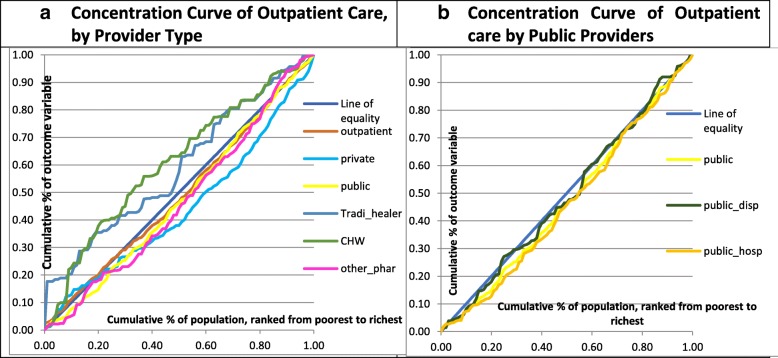
Concentration Curve of outpatient care. Source: ECVMAS 2013, using ADePT software

We estimated the CI and curve for outpatient services. Overall outpatient services are close to the line of equality with a CI of 0.02 (Table 4). Health care services provided by CHWs and traditional healers were identified as pro-poor based on their negative CIs of − 0.22 and − 0.18, respectively. In contrast, private facilities were found to be pro-rich with a CI of 0.12, followed by ancillary services at 0.07 CI. Public facilities have a positive CI, but very close to 0 as well (with a CI of 0.05). A breakdown of health care utilization by public facilities, public dispensaries and public hospitals found that public dispensaries are more strongly associated with pro-poor characteristics (CI of 0.02) than public hospitals (CI of 0.08). CI results are presented graphically in Fig. 3.

**Table 4:** Inequality of outpatient services, by provider type

	All health facilities (*N* = 1878)	Public health facilities (N = 806)	Public Dispensaries (*N* = 327)	Public Hospitals (*N* = 479)	Private for-Profit facilities (*N* = 472)	Ancillary Services (*N* = 274)	Community Health Workers (*N* = 112)	Traditional Healers (*N* = 104)
Inequality or Concentration Index (CI)	0.02	0.05	0.02	0.08	0.12	0.07	−0.22	− 0.18

Source: ECVMAS 2013 using ADePT software

#### Decomposition of the concentration index

The decomposition of CI by provider types presented in Table 5 shows that CHWs and traditional healers are the only providers concentrated among the poor. Among poor wealth quintiles, use of CHWs is more concentrated than use of traditional healers. However due to affordability-related (wealth quintiles) factors and demand, poor households are more likely to have small children, and parents of these children are more likely to seek care from CHWs. Traditional healers are also pro-poor, but this is mainly driven by availability (urban) and household size. The private sector is very pro-rich, and driven primarily by affordability. Public providers trend towards being pro-rich as well, however households with more children below age four tend to shift the CI of public facilities towards being more pro-poor. The following variations by type of public facility were identified: While wealth quintile, household size and gender make the CI of public dispensaries and hospitals trending pro-rich, households with more children below age four and having any level of education off-set this effect, making the CI more pro-poor, especially for public dispensaries. This effect seems marginal for public hospitals which are much more pro-rich than public dispensaries.

**Table 5:** Decomposition of the Concentration Index

	All health facilities (*N* = 1878)	Public health facilities (*N* = 806)	Public Dispensaries (*N* = 327)	Public Hospitals (N = 479)	Private for Profit facilities (*N* = 472)	Ancillary Services (N = 274)	Community Health Workers (*N* = 112)	Traditional Healers (N = 104)
Concentration index (Inequality)	0.02	0.05	0.02	0.08	0.12	0.07	−0.22	−0.18
Standardizing demographic variables								
Household size	0.02	0.02	0.03	0.00	−0.00	0.06	0.02	−0.04
Gender	0.00	0.00	0.00	0.00	0.00	0.00	0.00	0.00
Older than 65	−0.00	−0.00	−0.00	− 0.00	0.00	0.00	−0.00	− 0.00
< 4 years	−0.03	− 0.04	−0.07	− 0.02	−0.01	− 0.05	−0.07	− 0.01
Control variables								
Wealth quintiles	0.07	0.08	0.07	0.09	0.13	0.14	−0.07	0.07
Educated	−0.09	− 0.03	−0.03	− 0.02	−0.02	− 0.03	−0.03	− 0.04
Health Insurance	0.01	0.01	0.01	0.01	0.01	−0.00	0.03	0.00
Urban	−0.02	−0.00	− 0.02	0.01	0.01	−0.06	− 0.10	−0.13
Residual	0.01	0.02	0.03	0.01	−0.00	0.01	−0.00	−0.03

Source: ECVMAS 2013, using ADePT software

Methodological note: The decomposition of outpatient health services by provider type distinguishes the inequality measure from justifiable standardizing determinants such as age and gender- and unjustifiable determinants -the Z such as income, health insurance status. Each factor is drawn above or below zero– above 0 indicates a positive contribution of the factor making the CI more pro-rich and below 0 indicates a negative contribution of the factor making the concentration more pro-poor. The residuals show the part of the CI that is not due to the factors included in the analysis. In this study, gender and age and having children below 4 are seen as “need” variables that predict the need for health services, while wealth quintile, education, health insurance and residence as “non-need” variables, from which the differences of utilization resulted are considered as unfair and as inequity

#### Determinants of health seeking behavior

Econometric analysis confirmed the results of descriptive statistics, particularly in the area of socio-economic variables as key determinants of health care service utilization. As presented in Table 6, Individuals from the richest wealth quintile were three times more likely to use health care services when sick than were households from the poorest quintile (OR:3.07; *P* < 0.001), controlling for other variables. Individuals in the fourth wealth quintile^7^ were more likely to seek health care by 79% (OR:1.79; *P* < 0.01). Literacy also increased the likelihood of using health services by 63% (OR:1.63; P < 0.001). In contrast, geographic variables (e.g., living in a specific region or in an urban area) had no effect on health seeking behavior (Table 6). Therefore, demographic factors are considered to only play a marginal role in predicting health seeking behavior. In contrast, having an additional household member increased the likelihood of seeking health care services by 9% (OR:1.09; *P* < 0.05). Individuals with health insurance were eight times (OR: 8.12; P < 0.001) more likely to consult a health care provider when sick. Regression results of the health seeking behavior model are presented in Table 6.

**Table 6:** Regression results of health seeking behavior: Haiti, 2013 – individual level

	Odds Ratio (OR)	Standard Error (Std. Err)	z	95% Confidence Interval
Insurance (1 = having insurance; 0 = otherwise)	8.12***	4.82	3.52	1.40–9.45
Quintile (Poorest)				
Poorer	1.33	0.30	1.32	0.87–2.09
Middle	1.22	0.30	0.83	0.76–1.98
Richer	1.79**	0.46	2.28	1.09–2.95
Richest	3.07***	0.98	3.51	1.64–5.75
Having children < 4 y (yes = 1; otherwise = 0)	1.10	0.18	0.57	0.80–1.52
Having older > 65 y (yes = 1; otherwise = 0)	0.94	0.18	−0.33	0.64–1.37
Gender (1 = women; 0 = men)	1.09	0.19	0.51	0.78–1.55
Literate (1 = literate; 0 = otherwise)	1.63***	0.28	2.83	1.16–2.28
Urban (1 = living in urban area; 0 = rural area)	0.87	0.21	−0.56	0.54–1.40
Region (North)				
South	0.87	0.22	−0.52	0.53–1.45
Transversal	1.31	0.32	1.08	0.81–2.11
West	1.23	0.46	0.55	0.59–2.57
Metropolitan	0.63	0.18	− 1.58	0.36–1.12
Household size	1.09*	0.04	2.26	1.01–1.17
Constant	0.85	0.30	−0.46	0.42–1.70

Pseudo R2:0.051; Number of observations: 1534; Wald-Chi2: 56.87; * *p* < 0.05; ** *p* < 0.01, *** *p* < 0.001. Note: Std. Err. denotes standard error

#### Determinants of catastrophic health expenditures

The following paragraph describes results of the SUR model, including results of the chi square testing significance of variable differences over time (Table 7).

**Table 7:** Results of the seemingly unrelated regression of CHE: Haiti, 2012, 2013, household Level (end of document)

	2012		2013		Difference (2013–2012) Test (chi2)
	Odds Ratio (OR)	Standard Error (Std. Err)	Odds Ratio (OR)	Standard Error (Std. Er)	
Quintile (Poorest)					
Poorer	0.77	0.164	0.59*	0.12	0.79
Middle	0.83	0.183	0.42***	0.10	4.60*
Richer	0.72	0.181	0.30***	0.07	6.32*
Richest	*0.54	0.156	0.18***	0.06	6.01*
Having children < 4 y (yes = 1; otherwise = 0)	1.09	0.183	0.91	0.16	0.54
Literate (1 = literate; 0 = otherwise)	1.35	0.232	1.42*	0.25	0.04
Having older household > 65 y (yes = 1; otherwise = 0)	1.47*	0.257	2.04***	0.35	1.78
Gender (1 = women; 0 = men)	0.98	0.152	0.81	0.12	0.74
Household size	1.03	0.036	1.19***	0.04	9.30**
Region (North)					
South	1.54	0.38	1.25	0.31	0.34
Transversal	1.31	0.33	1.22	0.32	0.04
West	1.12	0.30	0.75	0.21	1.12
Metropolitan	0.95	0.24	1.19	0.31	0.37
Urban (1 = living in urban area; 0 = rural area)	1.04	0.22	1.19	0.25	0.19
Health system variables (2013)					
Health Insurance (yes = 1; otherwise = 0)			2.53*	1.19	
Public facilities (yes = 1; otherwise = 0)			3.83***	0.85	
Private facilities (yes = 1; otherwise = 0)			10.45***	2.47	
CHW (yes = 1; otherwise = 0)			0.29*	0.20	
Traditional Healer (yes = 1; otherwise = 0)			1.91	1.26	
Other and ancillary services (yes = 1; otherwise = 0)			1.08	0.53	
Constant	0.07	0.02	0.04	0.01	

Note: Each model had 2282 observations^a^. * *p* < 0.05; ** *p* < 0.01, *** *p* < 0.001

^a^The 2012 ECVMAS I had a sample size of 4,930 households which were representative at the department and national levels [15]. The 2013 ECVMAS II was a panel survey with a sample size of 2,282 households. These 2,282 households are the same households included in ECVMAS I’s larger sample of 4,930. The SUR model utilized these same 2,282 households from the 2012 and 2013 surveys to run the analysis

Wealth quintiles had a stronger influence on the rate of CHE in 2013 than in 2012. In 2012, the richest households were almost twice as likely not to face CHE compared to the poorest (OR:0.54; *P* < 0.05), but were 5.6 times less likely to experience CHE in 2013 (OR:0.18; *P* < 0.001). The change in the variable’s coefficient between 2012 and 2013 was found to be significant (Table 7). While the fourth and middle wealth quintiles had a lower probability of facing CHE than the poorest (first wealth quintile) in 2012, the relationship was not significant. Holding all other variables constant, the fourth wealth quintile was 3.4 times less likely to face CHE compared to the poorest (OR:0.30; *P* < 0.001), and the middle wealth quintile was 2.3 times less likely to face CHE in 2013 (OR: 0.42; *P* < 0.05). The values of coefficients of these two variables are significantly different between the 2 years. Poorer households (second wealth quintile) were less likely to incur CHE than the poorest (first wealth quintile) in 2012. This coefficient was not significant, yet became so in 2013 (OR: 0.59; P < 0.05). Test results were found not to be significant for the “poorer” (second wealth quintile) over time (Table 7).

Having a household member aged 65 or older was found to increase the odds of encountering CHE, with a higher OR in 2013 (OR: 2.04, *P* < 0.001) compared to 2012 (OR:1.47, P < 0.05). However, the difference between the values of the coefficient over time was not found to be significant. Gender and having children aged four or younger was not found to influence the rate of CHE in both years. Household size was found to influence the rate of CHE in 2013, but not in 2012. The relative number of household members (i.e., smaller to larger) increased the odds of facing CHE by 19% in 2013 (OR;1.19; P < 0.001) and the change in the variable’s coefficient between 2012 and 2013 was found to be significant. Households living in urban areas faced slightly higher odds of CHE than households living in rural areas in 2013, but the change in the variable’s coefficient between 2012 and 2013 was not significant. Overall, geographic location did not influence the rate of CHE.

The SUR models indicated that across the health system, having health insurance increased the likelihood of incurring CHE by 2.5 (OR: 2.53; P < 0.001) in 2013, holding all other variables constant. Surprisingly, households seeking care from public providers were almost four times more likely to incur CHE (OR:3.83; P < 0.001), while households seeking care from private facilities were 10 times more likely to incur CHE (OR:10.45; P < 0.001). In contrast, households seeking care from CHWs were 3.5 times less likely to incur CHE (OR:0.29; *P* < 0.05). Households going to traditional healers were more likely to incur CHE, but this relationship was not significant (Table 7).

## Discussion

This paper found out that the rate of CHE has increased between 2012 and 2013, particularly among the poorest wealth quintiles. The CI analysis underscored that public and private sector health services were not pro-rich, whereas CHW and traditional healers were pro-poor. The logit regression model on health seeking behavior in 2013 highlighted that individuals in the richest wealth quintile were three time more likely to use health services when sick than the poorest. Furthermore, SUR regression models on CHE in 2012 and 2013 found that wealth quintiles had a stronger influence on the rate of CHE in 2013 than in 2012. In 2012, the richest wealth quintile was almost twice as likely not to face CHE than the poorest wealth quintile but were 5.6 times less likely to experience CHE in 2013. This section discusses the outcomes of the CI analysis and regression models on health seeking behavior and CHE, both in Haiti and in comparison with other low-income countries (LICs) and low- and middle-income countries (LMICs). The discussion section is divided into two sections: The first section discusses results from the CI analysis; and the second section examines findings on the drivers of health seeking behavior and CHE.

### Concentration index

In Haiti, the high rate of CHE among the poor could stem from the absence of a pro-poor health system. This finding is clearly illustrated in the CI analysis. Despite a low positive coefficient, public health facilities remain pro-rich (CI of 0.05) and are associated with an increased risk of CHE (OR:3.83; *P* < 0.001). Inequities in access to health services at public facilities may be driven by public hospitals which have the highest positive CI among public facilities (CI of 0.08). In contrast, CI among public dispensaries is close to 0 (CI of 0.02). Overall, the positive association between public facilities and CHE (in the CHE SUR regression model, Table 7) may be related to OOP payments at points of service (e.g., outpatient user fees and drug-related costs) that all wealth quintiles, including the poor, incur on a continual basis. As shown earlier, outpatient user fees and the cost of medicines has been identified as a main driver of CHE in Haiti (Fig. 2), reflecting similar trends throughout the LAC region and other LICs and LMICs. For example, according to a 2018 study on financial protection looking at the drivers of CHE in LICs and LMICs, medicine costs are driving CHE in Guatemala, Sierra-Leone, Burkina-Faso, and Uganda; while outpatient user fees are a key driver of CHE in the context of outpatient care in Guinea, Bangladesh, and Liberia [23].

Unsurprisingly, private facilities are even more pro-rich than public facilities in Haiti and present an even greater risk to vulnerable populations of incurring CHE. This said, poorest wealth quintile households continue to seek health care services at private facilities. Additional research is needed to better understand why poorest wealth quintile households may be willing to risk accrual of significant personal debt in exchange for accessing privately provided health care services.

Results from the CI analysis in Haiti also mirror findings from other studies that have used the same methodology (e.g., estimating CI among public and private health facilities). A study on equity in health service utilization in Ghana, South Africa and Tanzania showed that both public and private health services were pro-rich [31]. As in Haiti, public health facilities were found to be less pro-rich in the three countries than private facilities. Tanzania had similar results to Haiti in that the CI of public health facilities was close to the equality line, yet remained pro-rich. In contrast, the CI of public health facilities was much higher in Ghana and South-Africa than in Haiti. In a separate study, poor population groups in Afghanistan used public facilities more frequently than wealthy populations, who tended to use private facilities instead [32]. In this same study, the CI of public facilities was negative [− 0.14] and truly pro-poor. In some LICs and LMICs, primary health care (PHC) facilities were found to be more pro-poor than public hospitals [33].

.In comparison to public and private health facilities, health care services provided by CHWs were found to be pro-poor in Haiti, with a negative CI of − 0.22. Households consulting CHWs were 3.5 times less likely to incur CHE. The literature from other LICs shows that services provided through CHWs has helped to expand the availability of health care coverage, while offering financial protections for the poor [34, 35]. Interestingly, seeking care from traditional healers was found to be pro-poor in Haiti (CI of − 0.18), yet was also associated with higher CHE (Table 7). Although this finding was not significant, it points to a concerning trend that the poor may be incurring high OOP payments to access tradition healer services without the benefits and protections of quality control in the delivery of these alternative services. More research is needed to better understand the profile of patients seeking health care services from traditional healers.

### Determinants of CHE and health seeking behavior

The increase of CHE rates between 2012 and 2013 for the poor, and the fact that poor are three time less likely to consult health care services when sick than the rich, suggests a potential explanation behind low UHC tracer coverage across the lowest wealth quintiles (Institut Haïtien de l’Enfance (IHE), ICF International 2018)). Such findings confirm that the national health system poses ever-growing inequities for the poor. The rate of CHE increased nationwide by 22% from 9.43% in 2012 to 11.54% in 2013, compared to an increase of 10% over a 10 year period throughout the LAC region (i.e., from 13.4% in 2000 to 14.8% in 2010 [9]).The rate of CHE increase was most notable among the poorest wealth quintile with an increase of 57% from 11.62% in 2012 to 18.20% in 2013. In contrast, the rate of CHE declined by 53% among the richest wealth quintile from 9.49% to 4.46% during the same period. These results mirror previous research conducted throughout LICs in which poor households were at higher risk of facing CHE than rich households. A 2011 study on the determinants of CHE in 12 Latin American countries found that poor households incurred higher rates of CHE using a 30% threshold of total consumption [36] than did rich households. A 2018 assessment of financial protection conducted by the Global Financing Facility (GFF) in 16 LICs and 8 LMICs found the rate of CHE by income quintile was more concentrated among the poorest groups [23]. A similar finding was observed in a 2017 assessment on CHE in LICs [37]. Continuing this trend, findings from a study on the determinants of CHE in Nigeria in 2015 showed that CHE rates were three times higher among lower income groups than among richer income groups [38]. In Senegal, Séne and Cissé (2015) also used SUR to assess the determinants and magnitude of CHE impact. Predictably, findings showed that the risk of CHE jeopardized household welfare, particularly among the poor [39].

National health accounts may give some insight as to the root causes of deteriorating financial protection for the poor between 2012 and 2013 ( [13]). There was a significant increase in OOP payments for health as a share of current health expenditures, shifting from 31% in 2012 to 40% in 2013 [13]. This increase coincided with a sharp reduction in external assistance, which decreased from 61% to 48% of current health expenditures over the same time-period [13]. In 2010, user fees were exempted across Haiti, but were reinstated in 2013 to compensate for the decline in external donor funding. Indeed, the 2013 Service Provision Assessment (SPA) confirmed that 94% of health facilities collected user fees in 2013 [40]. Additionally, the increase in household expenditures for medicines and medical supplies between 2012 and 2013 may be associated with decreased donor funding for the health sector (which includes disaster relief aid^8^) over these same years, and may have also contributed to increased rates of CHE.

Demographically, Haitian households with older members appear most vulnerable experiencing financial risk. This finding has also been observed in other LICs. For example, in Uganda households with elderly and unemployed family members were more likely to incur CHE [37]. Recognizing the cost of medicines as a key driver of OOP payments, we hypothesize that older populations in this study may have incurred debt due to the costs of medicines needed to treat non-communicable diseases.

While health insurance was positively associated with health service utilization in Haiti, it was also associated with CHE and may therefore not be a viable solution for preventing financial hardship among the nation’s poorest population groups. Similarly, access to health insurance may also push households towards an over-consumption of care without protecting them from financial hardship. Other LICs with health insurance have experienced similar deteriorations in CHE protections [9] [41] [42]. For example, the expansion of health insurance in the Philippines coincided with a worsening of financial protection for the poor because essential drugs were excluded from the national health care benefit package, resulting in a main driver of catastrophic spending [43]. In Haiti, national health insurance policies may also be a key driver of CHE because they do not adequately cover the costs of drugs. Additional research is needed to gain a better understanding of Haiti’s health insurance benefits package and its correlation with CHE among vulnerable populations.

## Study limitations

Limitations in this study present several challenges to internal validity due to its design as a quasi-experiment study. The model examining the determinants of utilization of health care services is based on one data point (i.e., 2013), though there could be several factors effecting utilization of health services over time. Notwithstanding this limitation, we offer findings from this study as a point of departure, recognizing similar analyses may be conducted using the results of future household surveys. The health seeking behavior model utilized also has weak explanatory power (R^2^ at 5%).

While financial barriers certainly pose an obstacle to accessing health services in Haiti, there are additional factors (e.g., cultural norms and traditions) which may also deter various populations from utilizing health services. Several qualitative studies have already highlighted the role of religion, voodoo, and other cultural considerations that influence health seeking behavior in Haiti. For example, a study examining the determinants of seeking care for mental health services in rural Haiti revealed that 32% of respondents selected God as their first choice of care, followed by clinics and hospitals [44]. While these considerations are important, the introduction of cultural factors does not dilute the main conclusions about inequalities presented in this study.

Finally, the absence of a control group in the study design introduces several limitations in the CHE model. Despite these limitations, we are confident of this study’s findings, as the data sources and methodology remained consistent over the two-year period examined.

## Conclusion and policy recommendations

By recognizing the right to health in Haiti’s constitution and making UHC a core objective of the 2012 National Health Policy, Haiti has committed to realizing UHC through a pathway that is consistent with universally recognized tenants of human rights. Findings from this study show that Haiti’s current approach to UHC expansion is being carried out in a pro-rich manner. Between 2012 and 2013, the rate of CHE among the richest wealth quintile decreased, while increasing sharply among the poorest wealth quintile.

Progress towards realizing the right to health in Haiti will require deliberate adjustments in national health care policies that incentivize health seeking behavior, while protecting the poorest wealth quintiles from the risk of CHE. We recognize that policy options are limited in a country such as Haiti due to highly constrained macro-economic conditions and low priority given to public health fiscal allocations (e.g., a decrease in per capita public health expenditures from USD $13 million in 2000 to USD $7 million in 2015 (Tandon A., et al., unpublished observations^9^)). Yet donor funding from the international community continues to fuel a substantial share of Haiti’s public health financing (i.e., 49% in 2015 [13]) and can be repositioned to prioritize pro-poor interventions.

Recognizing the resource, administrative and data constraints inherent in Haiti’s health sector, we offer the following pro-poor policy recommendations for the Government of Haiti and its health partners to consider. These recommendations prioritize interventions that would alleviate the burden of health care costs, while introducing sustainable incentives to increase health seeking behavior among Haiti’s poor and marginalized populations.

1. **Expand access to CHW-provided health care services in geographic areas with little to no existing coverage.** Given the pro-poor nature of health care services delivered by CHWs, expanded coverage would strengthen access to preventive health care and promote a more robust referral system among poor households. Expanded CHW coverage would also lower the risk of CHE among vulnerable populations who would otherwise be deterred from seeking necessary care. To maximize resources and efficiencies, the strategic expansion of CHW services can be integrated into existing priority health care programming (e.g., for cholera or malaria).
2. **Reduce the costs of medicines and medical supplies in public dispensaries, health centers and community hospitals through subsidies and more efficient supply chain management systems.** Approximately 70% of CHE is associated with the costs of medicines and medical supplies. Addressing this vulnerability through national policies that explicitly reduce the cost of said public health commodities is essential. This can be achieved through procurement policies that favor less expensive drugs and generics, and by increasing the availability of drugs in public health facilities and dispensaries where the poorest tend to seek care. Reducing the cost of medicines and supplies through updated national procurement regulations, targeted pro-poor subsidies and prioritization of supply chain enhancements that minimize leakage of subsidized commodities will go a long way towards achieving more equitable and affordable access to health care among poor and vulnerable segments of the population.
3. **Reduce CHE by lowering user fees at outpatient points of service, especially in pro-poor public facilities and dispensaries, through Haiti’s Results-based Financing (RBF) program**. Initiated in 2016, Haiti’s RBF program aims to improve service utilization and quality of care by providing financial incentives to facilities and providers at the primary care level based on performance (e.g., quantity and quality of services provided). Reducing the rate of CHE may be achieved by introducing a policy of reduced user fees within the existing RBF program which is currently being scaled up in more than 200 health facilities across the country. Since the RBF program operates at the primary care level (including in public dispensaries), this type of policy would benefit poorer households.
4. **Explore a pro-poor reorientation to disaster relief programming.** Haiti is prone to national disasters. This study demonstrates that the poorest wealth quintiles are disproportionately affected when external assistance is withdrawn. Future research should prioritize understanding the root causes of this phenomenon and suggest evidence-based interventions that can mitigate this inequity in a locally sustainable manner.
5. **Reexamine coverage offered in the existing health insurance package:** Existing health insurance mechanisms increase the rate of CHE. An updated examination of the types of services covered by health insurance and drug reimbursement policies is warranted to improve coverage and reduce costs among Haitians. Given that health insurance is primarily attained only by rich wealth quintiles, the expansion of health insurance coverage to poor wealth quintiles will be an important component in addressing overall health care inequities nationwide.

As demonstrated in the 2017 World Bank report [14], there are several entry points within the Haitian health system where efficiencies may be gained. These include improved donor-government coordination in the area of annual resource allocations and public sector financial management reforms. Said gains in efficiency may provide an important source of revenue that would be required to implement the recommendations offered in this study. While this and other potential sources of funding may be helpful, achieving the right to health for all Haitians will require the will to allocate financial resources in an equitable manner, and substantial political commitment at the highest levels of government and society.

## Abbreviations

ADePT: Automated DEC Poverty Tables
CHE: Catastrophic Health Expenditures
CHW: Community Health Worker
CI: Concentration Index
DECRG: Development Research Group (World Bank)
DHS: Demographic Health Survey
ECVMAS: Enquête sur les Conditions de Vie des Ménages Après Séisme
GFF: Global Financing Facility
HTG: Haitian Gourdes
IHE: Institut Haïtien de l’Enfance
LAC: Latin America and Caribbean (region)
LIC: Low-Income Country
LMIC: Low- and Middle-Income Countries
MMR: Maternal Mortality Ratio
OLS: Ordinary Least Square
OOP: Out of Pocket (payments)
OR: Odds Ratio
PHC: Primary Health Care
RBF: Results-based Financing
SCI: Service Coverage Index
SD: Standard Deviation
SDG: Sustainable Development Goal
SPA: Service Provision Assessment
Std Err: Standard Error
SUR: Seemingly Unrelated Regressions
TBA: Traditional Birth Attendant
THexp: Total Household Expenditures
U5MR: Under 5 Mortality Rate
UHC: Universal Health Coverage
WB: World Bank
WHO: World Health Organization

## Footnotes

1 The 2012 ECVMAS I survey did not include data on morbidity or health seeking behavior.

2 Health services which are primarily used by the poor wealth quintiles (e.g., the first and second wealth quintiles) are considered pro-poor. In contrast, health services used by the rich quintiles (e.g., the fourth and fifth wealth quintiles), considered pro-rich.

3 ADePT is an automated economic analysis software platform developed by the WB’s Development Research Group (DECRG). ADePT uses micro-level data from various types of surveys (e.g., Household Budget Surveys, DHS and Labor Force surveys) to produce rich data sets in various areas of economic research. ADePT contains a health module which estimates concentration curves and indices of health services based on household surveys.

4 As indicated in data source and sampling, health seeking behavior-related questions were only reported in the 2013 ECVMAS II survey. This study therefore only analyzed the drivers of health seeking behavior for 2013.

5 In 2012, 1USD = 42 Haitian Gourdes. In 2013, 1USD = 44 Haitian Gourdes [24, 25]

6 The 2013 ECVMAS II includes a sample of 2282 households, while the 2012 ECVMAS I includes a sample of 4930 households. When conducting the SUR model, the regression utilizes the same 2282 households identified in both the 2012 and 2013 surveys to ensure parity between the 2012 and 2013 equations.

7 Consumption quintiles are used to distinguish population groups according to their economic welfare: poorest households are grouped together into the 1st quintile, those with higher consumption into the 2nd quintile, and so on. Five quintiles rank the population from the poorest 20% to the richest 20%. The purpose of wealth quintile designations is to analyze how social and economic indicators change in relation to a population group’s welfare status [30].

8 Following the earthquake of 2010, international donor funding for the health sector jumped from covering 43% of current health expenditures in 2010 to covering 65% of current health expenditures in 2011 [12]. This additional significant increase in donor funding for the health sector was driven by the devastating earthquake in 2010 and emphasized disaster relief aid.

9 Tandon Ajay, Cain Jewelwayne, Kurowski Christoph, and Postolovska Iryna (2018), Cross-Country Public Financing Dynamics: Fiscal Space Accounting for Health, Draft HNP Discussion Paper, World Bank, Washington, DC

## Authors’ information

The findings, interpretations, and conclusions expressed in this paper are entirely those of the authors as individuals and should not be attributed in any manner to the institutions to which each author is affiliated.
